# Spatial Coding as a Function of Handedness and Responding Hand: Theoretical and Methodological Implications

**DOI:** 10.1371/journal.pone.0151979

**Published:** 2016-03-31

**Authors:** Isabel Arend, Peter H. Weiss, David C. Timpert, Gereon R. Fink, Avishai Henik

**Affiliations:** 1 Department of Psychology and the Zlotowski Center for Neuroscience, Ben-Gurion University of the Negev, Beer-Sheva, Israel; 2 Cognitive Neuroscience, Institute of Neuroscience and Medicine (INM-3), Research Centre Juelich, Juelich, Germany; 3 Department of Neurology, University Hospital Cologne, Cologne, Germany; University Medical Center Goettingen, GERMANY

## Abstract

The Simon effect shows that choice reactions are faster if the location of the stimulus and the response correspond, even when stimulus location is task-irrelevant. The Simon effect raises the question of what factors influence spatial coding. Until now, the effects of handedness, responding hand, and visual field were addressed in separate studies that used bimanual and unimanual tasks, providing inconclusive results. Here we aimed to close this empirical gap by looking at the effects of these variables in the same study. We used a unimanual version of a Simon task with four groups of participants: left-handed and right-handed, responding with the dominant or nondominant hand. Our results show that the Simon effect is substantially reduced in the field of the responding hand for all groups of participants, except for left-handed individuals responding with the left-hand. These findings highlight the importance of attention mechanisms in stimulus-response coding. They reflect that stimulus-response interference is influenced by hierarchical activation of response units. At a practical level, these findings call for a number of methodological considerations (e.g., handedness, responding hand, and visual field) when using stimulus-response conflict to address spatial coding and cognitive control functions in neurological populations.

## Introduction

In order to efficiently perform spatially directed actions, many organisms need to integrate visual information presented in different spatial locations with an appropriate motor plan. The response to different traffic lights or signs is an example of an individual’s need to associate different visual input with different responses. In the laboratory, the influences of spatial processing on response have been studied using the Simon task [1]. In a typical Simon task, participants are asked to respond using their left and right hands to two different stimuli features (e.g., red and green colors). In every trial, a red or a green target appears at the left or right side of a fixation point. Although the location of the stimulus is task-irrelevant, it significantly affects an individual’s response. That is, when the location of the stimulus and the response correspond (Simon compatible trial) response times (RTs) are faster than when the location of the stimulus and the response do not correspond (Simon incompatible trials).

Accounts for the Simon effect are based on dual-route models in which it is assumed that the appearance of the stimulus automatically activates the associated response [2,3]. In dual-route models, one component reflects the priming of a spatially corresponding response by the abrupt stimulus onset. The second component reflects the task-defined transformation of relevant stimulus information to spatial codes. The Simon effect has been used extensively in both behavioral and neuroimaging research to study the neural basis of cognitive control and different aspects of perception and action coding [4,5].

The fact that spatial locations cannot be ignored in this task raises a question regarding the factors that influence spatial coding. It has been shown that an individual’s handedness coupled with his/her responding hand are important factors influencing the way spatial coding takes place because they are related to how attention is allocated across space [4,6,7]. The present work investigates the role of handedness and responding hand in a unimanual version of the Simon task in which participants were required to decide the direction of motion of a flowfield of moving dots.

### Spatial Coding in the Simon Task

There have been two prominent hypotheses accounting for the formation of spatial codes in the context of the Simon task: the *attentional hypothesis* and the *referential coding* hypothesis. The attentional hypothesis was first outlined by Simon himself [8]. Simon explained the effect as a natural tendency to respond to the source of a stimulus. This tendency is analogous to a reflexive shift of attention toward a stimulus. The attentional account for the Simon effect is grounded on the idea described in the Premotor Theory of Attention (PMTA) [9] that spatial attention and the motor system are coupled. According to the PMTA, shifts of spatial attention are equivalent to the preparation of an eye movement to a target’s location [9]. Therefore, the abrupt onset of the stimulus in the visual periphery in the Simon task produces a shift of attention that facilitates the selection of a motor response.

The role of attention in the Simon effect was examined in a bimanual version of the Simon task in response to stimuli at various spatial locations [10]. A visual cue was presented at different spatial locations before the target. The logic behind this manipulation was that if attention was oriented toward a particular point in space, before the presentation of the stimulus, that point would be the reference to code the stimulus position as “left” or “right.” Results showed that left-hand responses were faster for stimuli presented on the left side of the focus of attention (cued location), and the same pattern was found for right-hand responses. These findings were interpreted in terms of left and right sides of space being coded in reference to the location of the focus of spatial attention. Later, a number of reports provided additional evidence for the fact that shifts of attention are an important component in generating the Simon effect [11–17].

The *referential coding hypothesis* holds that the Simon effect is the result of spatial representation and response codes that are not dependent on absolute spatial locations [18,19]. It is important to note that the referential coding hypothesis makes clear that the stimulus and response codes are formed with respect to one or more objects simultaneously [5]. For example, even though participants respond with the two fingers of the same hand, a Simon effect is expected. This is due to a correspondence between “left and right” response and “left and right” location codes. Although this account does not rule out some influence of attention, it holds that spatial coding is *not a result* of attentional shifts, but rather, of how response and spatial codes are represented.

The *grouping model* was developed to account for the role of the effectors’ location on spatial coding [20,21] and was originally not part of the debate concerning the Simon effect. However, this model speaks closely to the issue regarding possible effects of the stimulus and effectors’ location. The model describes how stimuli and response units are represented in functionally different cognitive representations that use multiple and hierarchical coding schemes. The grouping model was developed using response cueing paradigms in which individuals were required to respond to visual stimuli using a four-choice finger setup [22]. A visual cue signaling two out of four response options was presented before the target so that participants could prepare their response. The cues could signal two fingers of the same hand or two fingers of different hands. Results from these tasks show a benefit for *same hand* over *different hand* conditions. The same-hand advantage is explained in terms of a preattentive grouping process of response units. That is, visual input elements and response elements are not processed independently, but rather, are grouped according to low-level factors, for example, by Gestalt principles. This account clearly predicts that the location of the effectors and the location of the stimulus should have an impact on the Simon effect.

### Effects of Handedness, Responding Hand, and Visual Field in the Simon Task

In stimulus-response compatibility effects, such as the Simon task, handedness, responding hand, and visual field are critical variables to be taken into account when studying stimulus-response coding. To examine these factors, the Simon effect needs to be analyzed as a function of visual field and responding hand [23,24]. However, except for the few studies described here, researchers computed the Simon effect by averaging left and right target response times and visual field. Therefore, these studies do not allow for an examination of asymmetries in the Simon effect as a function of these variables. The few studies that have analyzed visual field and responding hand provide empirical support for the suggestion that these variables influence spatial coding and that a systematic investigation that includes these variables into the same experimental design is needed.

In a unimanual Simon task [25]—for stimulus-response compatibility [26]—left and right responses were mapped to the index and middle fingers of the same hand [25]. Across different blocks, right-handed participants were asked to respond with their left or right hand. If spatial coding is dependent on the location of the responding hand (because attention is drawn to the effector hand), an asymmetrical Simon effect should be found. Alternatively, if space is coded in abstract and not in absolute terms, the Simon effect should be symmetrical for left and right stimuli, irrespective of the location of the responding hand. In three experiments, participants performed the Simon task under palm up and palm down conditions, and the responding hand was alternated or fixed within the experimental sessions. A symmetrical Simon effect was found under the “alternated” condition. However, when hand position was fixed within the block, left-hand responses were significantly slower than right-hand responses. Interestingly, there was a trend for a smaller Simon effect for right-side stimuli versus left-side stimuli. It is important to note that this study reported on a small group of right-handed participants (eight in total). Therefore, it is possible that failure to find a significant effect of responding hand and target location was due to lack of statistical power.

Two studies looked at the effects of handedness and responding hand as a function of visual field in bimanual tasks. In one of the studies, the performance of right- and left-handed individuals was analyzed in an auditory Simon task [27]. An asymmetrical Simon effect was observed: The effect was significantly larger in the right hemifield for right-handers and in the left hemifield for left-handers. Later, the Simon task was conducted in three groups of individuals: left-handed, ambidextrous, and right-handed [6]. Results once more showed an asymmetrical Simon effect across the left and right visual field. The Simon effect was larger in the field where the dominant hand was operating; in other words, the effect was larger on the right side for right-handed participants and on the left side for left-handed participants. The ambidextrous group showed a symmetrical pattern. Authors interpreted these findings in terms of hand-centered attentional bias [6]. In line with the interpretation offered previously [25,26], spatial attention was shown to be influenced by constant spatial cues, that is, by the location of the dominant hand.

A computational model on right-hand asymmetries on the Simon effect was formulated based on the performance of right- and left-handers in a bimanual Simon task [7,27]. Results showed a smaller Simon effect on the left side for right-handers and a reverse Simon effect for left-handers. These asymmetries were interpreted to reflect left-right hemispheric specialization for both orienting of attention and response selection. This view is consistent with the attention account for the Simon effect. The referential coding account was discarded as an explanation for these asymmetries [18]. That is, there was no reason to expect an advantage of the corresponding response on one side but not on the other.

Taken together, these findings can be summarized as follows: 1) under bimanual response conditions, when handedness and visual field are taken into account, an asymmetrical Simon effect is found. That is, the Simon effect is larger in the field of the dominant hand for both left- and right-handed individuals; 2) under unimanual response conditions, right-handed individuals show a trend for a smaller Simon effect in the right visual field. Therefore, the results for the unimanual tasks are not conclusive. In general, the results showing an asymmetrical Simon effect (e.g., for bimanual tasks and partly for the unimanual tasks) are in agreement with the attentional hypothesis. However, the results for unimanual tasks do not provide any information on whether the subtle reduction of the Simon effect in the right visual field is derived by participant’s handedness or by the manipulation of the responding hand. This inconclusive finding again stresses the necessity that handedness, responding hand, and visual field should be used as factors within the same experiment.

### The Present Study

The basic idea underlying the referential coding hypothesis is that the Simon effect arises as a consequence of how spatial and response codes are cognitively represented. Therefore, the best way to test this hypothesis is to use tasks that imply some kind of relative coding process, for example, by using unimanual responses in which left and right keys are associated with two fingers of the same hand. Given the importance of the position of the effectors for the attentional hypothesis and for the grouping model, the use of unimanual responses should be combined with an individual’s handedness and responding hand so that the impact of hand dominance on spatial coding can be evaluated.

Accordingly, we tested four groups of participants: left-handed individuals responding with the dominant (left) hand, left-handed individuals responding with the nondominant (right) hand, right-handed individuals responding with the dominant (right) hand, and right-handed individuals responding with the nondominant (left) hand. Moreover, left and right responses were mapped with the left and right fingers of the same hand.

The referential coding hypothesis allows clear predictions regarding the effects of handedness and responding hand as a function of visual field in the Simon task. According to the referential coding hypothesis, relative location of the stimulus and responses are translated into a spatial code; therefore, an advantage (faster responses) is expected whenever stimulus and responses share similar codes. That is, compatible trials should be faster than incompatible trials, independent of which hand is used for responding. This account predicts a symmetrical Simon effect across visual field and responding hand.

The attentional hypothesis predicts that any factor influencing orienting of attention has the potential to affect spatial coding. Consistent with the idea that spatial attention and motor systems are coupled, spatial shifts of attention are considered to influence stimulus-response correspondence. According to previous work [6,7,25], the location of the dominant hand acts as a constant spatial cue that biases an individual’s attention. Indeed, it was found that the Simon effect was asymmetrical in the location where the dominant hand was operating [6,7]. If an attentional bias produced by the location of the dominant hand influences spatial coding, we should find an asymmetrical Simon effect when participants respond with their dominant hand. The Simon effect should be larger in the field of the dominant hand [6].

We previously introduced the grouping model [20,21] to describe the potential impact of the location of the responding hand on the Simon effect. According to this model, preattentive grouping processes involving different response units may pose an advantage when the stimulus activates two associated responses. Therefore, this model predicts an asymmetrical Simon effect in unimanual experimental setups, independent of responding hand. In a unimanual response setting like the one used here, left and right responses were given with left and right fingers of the same hand, and therefore, the two fingers were part of the same response unit. Following the grouping model, if a participant responded with the right hand, the presentation of a target in the right visual field would automatically activate both response units (left and right fingers of the right hand) because they belong to the same response group (i.e., the right hand). Therefore, the mismatch between stimulus location and response location that characterizes the incompatible Simon effect should be substantially reduced in the field of the responding hand.

To summarize, our study has both theoretical and methodological implications. At a theoretical level, studying the effects of handedness, responding hand, and visual field provides a window into the role of the effectors’ location on spatial coding in line with the models presented earlier. At a methodological level, understanding the effects of response on the Simon effect is fundamental when using the Simon task to address the neural basis of perception and action coding in neurological populations. Patients diagnosed with both cortical and subcortical lesions are often unable to respond using their contra-lesional hand [28]. Therefore, the study of asymmetries produced by response-related factors is important when interpreting visual field effects observed in neurological patients. Previous studies using the Simon effect in neurologically healthy individuals assumed that the Simon effect was symmetrical between left and right visual fields in unimanual response setups.

## Method

### Participants

Thirty-two right-handed (19 female) and 32 left-handed (18 female) university students received course credit in exchange for participating in the present study. Handedness was assessed according to the Edinburgh handedness inventory [29]. All participants had normal or corrected-to-normal visual acuity and were neurologically healthy. The mean age was 21 years (*SD* = 2.7 years). We only included participants with a manual preference of +90 to +100 (right-handed) and -90 to -100 (left-handed). The present study was approved by the Ethics Committee of Ben-Gurion University of the Negev. All participants provided written consent to participant in the study. After the experiment, participants were debriefed.

### Apparatus and stimuli

**Fig 1:**
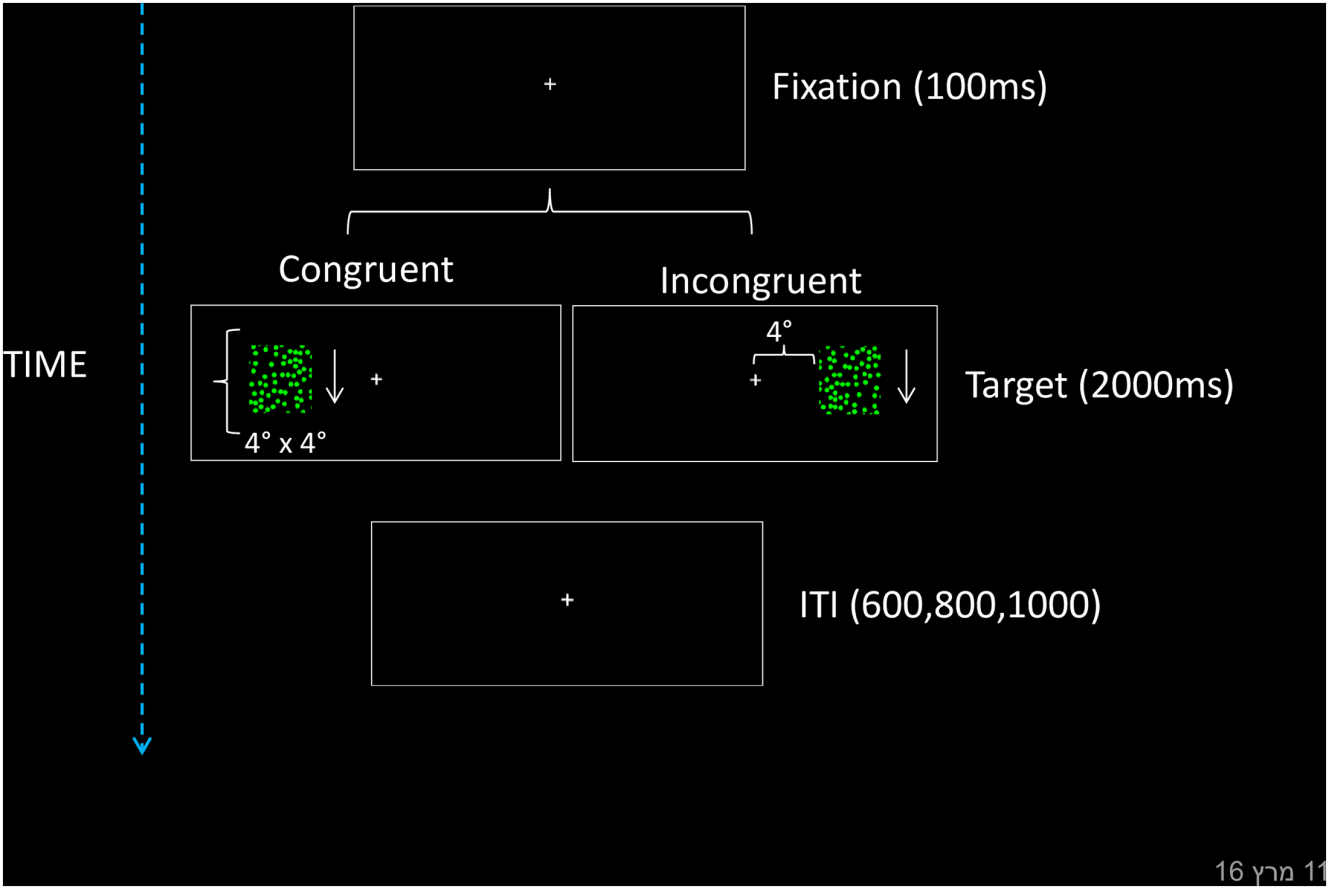
Layout and timing of the task. In different trials, a flowfield of moving dots was presented in the left or right visual field. Motion direction was mapped with left and right responses. Following the example given here, when a downward motion target is mapped with left responses and the target appears in the left visual field, the trial is compatible; when upward motion appears in the left field the trial is incompatible.

The experiment took place in a dimly light room and was programmed in E-prime [30]. Stimuli consisted of a flowfield of moving dots. Two patches of flowfields of upward and downward motion were used as targets. The flowfield density was set at 0.0075 dots/pixel^2^. The dots were randomly distributed within a square subtending 4° x 4° of visual angle from a viewing distance of approximately 60 cm. The boundaries of the square were not visible to the participants (see Fig 1). The dots moved at a speed of 45 pixels per second.

### Procedure

Each trial started with a central fixation cross that remained on the screen throughout the trial (see Fig 1) and which subtended a visual angle of ∼.07° x .07° (from a viewing distance of approximately 60 cm from the screen). This initial size was presented for a period of 1,000 ms, after which a change in size (∼.05° x 0.5°) for 500 ms signaled the start of the trial. One of two patches of moving dots (downward or upward) appeared randomly on the left or right side of the fixation cross (eccentricity was ∼.04° of the visual angle). The patch was presented until response or for a total period of 2 seconds. If no response was given, the trial started again. The intertrial interval randomly varied from 600–800 ms. Participants were required to respond as quickly as possible, while avoiding mistakes, to the direction of motion of the moving dots by using left or right keys of a response box. We used E-prime responses boxes. The inter-key distance was 1 cm. Participants responding with the left hand had the response box positioned on the left side of the body midline. Participants responding with the right hand had the response box positioned on the right side of the body midline.

Motion direction (downward and upward) and response key (left vs. right) were counterbalanced across participants. Participants used the index and the middle fingers of the same hand to respond. The responding hand was always positioned to the right (for the right-hand response condition) or to the left side (for the left-hand response condition) of the body midline.

The experimental design consisted of two between-subject factors of handedness (left-handed individuals and right-handed individuals) and responding hand (dominant and non-dominant), and two within-subjects factors of compatibility (compatible and incompatible) and visual field (left and right). Each participant completed one practice block consisting of 12 trials and two experimental blocks of 80 trials each (80 trials in each visual field; 40 compatible and 40 incompatible), resulting in a total of 160 experimental trials. Participants received feedback during the practice block. The experiment took approximately 20 minutes to complete. Once the session was terminated, participants were debriefed.

## Results

### Accuracy

We performed a mixed analysis of variance (ANOVA) on mean accuracy, with compatibility (compatible, incompatible) and visual field (left, right) as within-subject factors, and responding hand (left, right) and handedness (left, right) as between-subject factors. There was a significant main effect of compatibility, *F* (1, 60) = 18.03, *p* < .001, *η2p =* .231, reflecting that accuracy was higher for compatible (.98) versus incompatible trials (.95). The interaction between compatibility and handedness was also significant, *F* (1, 60) = 9.67, *p* = .003, *η2p* = .139. Planned comparisons revealed that left-handed participants were significantly more accurate for compatible (.98) versus incompatible (.94) trials, *t* (31) = 6.30, *p* = .0001; whereas the right-handed participants did not show a significant difference between compatible versus incompatible trials (.97 vs. .96, respectively, *t* < 1).

### Response times

Analysis of RTs was performed for correct trials only. RTs from 150 ms to 1,500 ms were included in the analysis. This procedure removed less than 2% of the trials. We ran a mixed ANOVA on mean RTs, with compatibility (compatible, incompatible) and visual field (left, right) as within-subject factors and responding hand (left, right) and handedness (left, right) as between-subject factors. There was a significant main effect of compatibility, *F* (1, 60) = 98.07, *p* < .001, *η2p* = .620; RTs for compatible trials were faster (540 ms) than for incompatible trials (588 ms), revealing the typical Simon effect.

**Fig 2:**
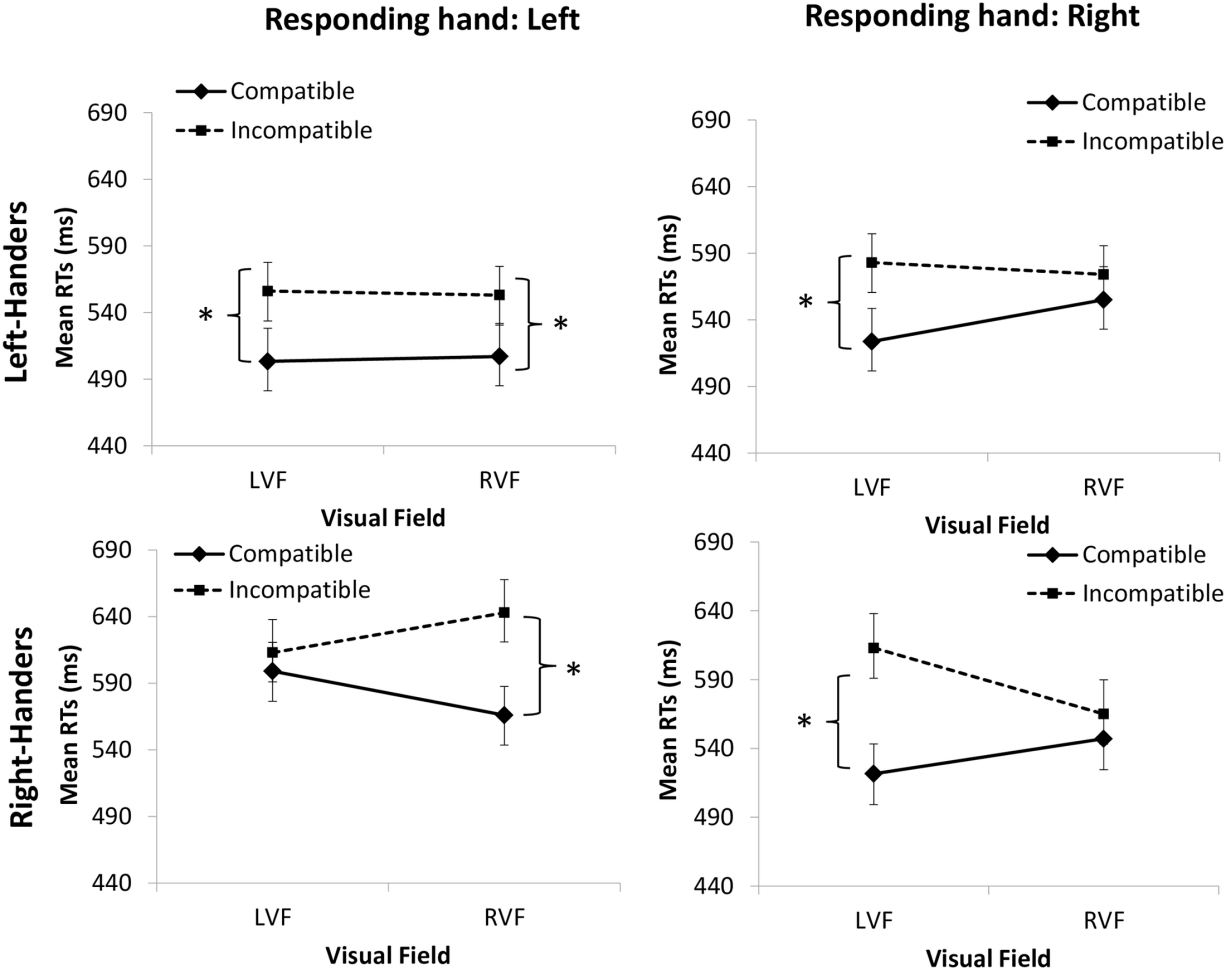
Each panel shows mean RTs as a function of compatibility (compatible and incompatible) and stimulus locations (left and right visual field; LVF and RVF, respectively). Compatibility is defined by the relationship between response and stimulus location; when response side (e.g., left) corresponds to visual field (e.g., LVF), the condition is compatible, and when response side and visual field do not correspond, the condition is incompatible. The responding hand was always positioned to the right (for the right responding hand condition) or to the left (for the left responding hand condition) of the body midline. Panels A and B show results for left-handers for the left and the right responding hand, respectively. Panels C and D show results for the right-handers for the left and right responding hand, respectively. Note that a symmetrical Simon effect was only found for the group of left-handers responding with the left hand (Panel A). Error bars represent standard error of the mean. Stars indicate significant effects (*p* < .01).

Of main relevance for the present report is that the four-way interaction reached significance, *F* (1, 60) = 4.70, *p* = .034, *η2p =* .73, and that the three-way interaction involving compatibility, visual field, and responding hand was also significant, *F* (1, 60) = 12.58, *p* < .001, *η2p =* .173. We decomposed the four-way interaction by conducting two 3-way ANOVAs separately for the two left-handed groups (see Fig 2, Panels A and B, respectively) and for the two right-handed groups (see Fig 2, Panels C and D, respectively). Compatibility and visual field were within-subject factors, and responding hand was a between-subjects factor. For the left-handed group, only the main effect of compatibility reached significance, *F* (1, 30) = 52.53, *p* < .001, *η2p* = .64 (see Fig 2). However, the results depicted in Fig 2 (Panel A and B, respectively) represent an asymmetrical Simon effect for the left-handed group responding with the right hand, but not for the left-handers responding with the left hand. In order to further explore this pattern, we conducted two-way ANOVAs taking compatibility and visual field on mean RTs separately for each group. For the left-handed group responding with the left hand only, the main effect of compatibility reached significance, *F* (1, 15) = 47.16, *p* < .001, *η2p =* .78, but not the interaction between the two factors, *F* (1, 15) = 0.12, *p* = .731, *η2p =* .008. For the left-handed group responding with the right hand, again only the main effect of compatibility reached significance, *F* (1, 15) = 15.75, *p* < .001, *η2p =* .52, but not the interaction involving compatibility and visual field, *F* (1, 15) = 1.36, *p* = .25, *η2p =* .083. Considering the pattern depicted in Fig 2 (Panel B), it is possible that the lack of significant effect might be due to low statistical power. Therefore, we examined the Simon effect (compatible vs. incompatible) for left-handers responding with the right hand by means of paired *t*-tests separately for the left and right visual fields. A significant Simon effect was observed for the left, *t* (15) = 2.85, *p* = .021, but not for the right visual field, *t* (15) = 1.07, *p* = .29.

Results for the two groups of right-handed individuals showed a significant main effect of compatibility, *F* (1, 30) = 47.00, *p* < .001, *η2p* = .61, and a significant three-way interaction of compatibility x visual field x responding hand, *F* (1, 30) = 21.11, *p* < .001, *η2p* = .41. We further explored this three-way interaction with two separate ANOVAs, with compatibility and visual field as factors, for the right-handed group responding with the right hand and for the right-handed group responding with the left hand. For the right-hand group responding with the right (dominant) hand (see Fig 2, Panel D) there was a main effect of compatibility, *F* (1, 15) = 21.85, *p* = .001, *η2p* = .59, and also a significant interaction between compatibility and visual field, *F* (1, 15) = 13.95, *p* = .002, *η2p* = .48, reflecting the asymmetry between left and right visual field Simon effects. That is, the interaction revealed that the compatibility effect was significant for the left visual field, *t* (15) = 5.31, *p* = .001, but not for the right visual field, *t* (15) = 1.16, *p* = .27. Similar effects were found for the right-handed group responding with the left (nondominant) hand (see Fig 2, Panel C). There was a significant main effect for compatibility, *F* (1, 15) = 25.19, *p* = .001, *η2* = .63, and also for the interaction of compatibility and visual field, *F* (1, 15) = 9.11, *p* = .009, *η2p* = .38. The interaction indicated that the compatibility effect was significant for the right visual field, *t* (15) = 5.96, *p* = .001, but not for the left visual field, *t* (15) = 1.10, *p* = .29. Taken together, these results suggest that the Simon effect was reduced in the field of the responding hand for the right-handed group (Fig 2, bottom panels).

### Exploring possible effects of finger superiority

Consistent with the logic used to examine effects on stimulus-response association, in the present study we used the factor of “compatibility.” However, it is possible that finger superiority (e.g., faster responses with the index than the middle finger) may have played a role in the effects reported here. To examine this issue, we re-coded the factor compatibility into visual field and finger (index and middle). We conducted a mixed ANOVA on mean RTs taking visual field (left vs. right) x finger (index vs. middle) as within-subject factors and handedness (left vs. right) and responding hand (dominant vs. non-dominant) as between-subject factors. We found a significant main effect of finger, *F* (1, 60) = 12.58, *p* < .001, *η2p* = .17; RTs were faster for the index (533 ms) as opposed to the middle finger (575 ms). However, there was also a significant 3-way interaction involving visual field x finger x responding hand, *F* (1, 60) = 98.07, *p* < .001, *η2p* = .62. That is, for individuals who responded with the left hand, index finger responses were faster for the right visual field than for the left visual field stimulus, *t* (31) = 6.47, *p* < .001. When individuals responded with the right hand, the pattern was reversed. That is, index finger responses are faster when the stimulus appeared in the left visual field as opposed to when the stimulus appeared in the right visual field, *t* (31) = 5.56, *p* < .001. These results show that the effects reported here cannot be attributed to the superiority of index finger responses.

## Discussion

In the present study we re-evaluated the role of handedness and responding hand in spatial coding by using a unimanual version of the Simon task. The Simon effect across left and right visual fields was analyzed for two groups of right-handed individuals: a group responding with the right (dominant) hand and a group responding with the left (non-dominant) hand, and for two groups of left-handed individuals under the same conditions. Results were unambiguous for the right-handed group: 1) the Simon effect was found to be asymmetrical across left and right visual fields, that is, the compatibility effect was smaller on the side of the responding hand; and 2) for the left-handed group, the asymmetry was found only for the group responding with the nondominant hand, but not for the group responding with the dominant hand.

### The role of attention in the Simon effect

Given the results for the right-handers, the effects of handedness and responding hand on spatial coding reported here speak to the general issue of orienting of attention [10]. Directing attention to a position in space brings about a right-left perceptual organization of the stimulus display, including the relevant and irrelevant elements of the task. That is, the location of the responding hand acts as a cue that produces an imbalance in the allocation of attention [25]. In general, our results are in agreement with the attentional account for the Simon effect, and they support the general link between attention and the motor system outlined by the PMTA [9].

The referential coding hypothesis cannot account for our results without considering the role of attention in the formation of spatial codes. The basic idea outlined by the referential coding hypothesis is that stimuli and responses are represented in relative terms. Therefore, the location of the responding hand should not have any impact on spatial coding as long as responses are defined in terms of left and right. Our results do not support this prediction because the Simon effect was substantially reduced in the field of the responding hand (for three of our four experimental groups).

Before we outline an explanation for the results of the left-handed individuals, let us consider the lateralization of attention, motor functions, and handedness. First, the asymmetrical Simon effect observed for left-handers using the nondominant hand does not seem to be due to hemispheric differences in attention functions. Spatial attention functions have been shown to be lateralized to the right hemisphere in right-handed individuals [31,32]. A large degree of left-hemisphere lateralization for spatial attention has also been reported for left-handers [33]. Any explanation evoking differences in spatial attention alone between left and right hemispheres needs to account for the pattern of right-handers under the same conditions.

Second, any account for the symmetrical Simon effect for the left-handers responding with the dominant hand requires that both hemispheric and responding-hand activation effects should be taken into consideration. Generally speaking, the results for the left-handed group are in agreement with the observation that left-handed individuals are found to be less asymmetrical than right-handed individuals in manual tasks [34,35]. This smaller behavioral asymmetry is in accord with the reduced asymmetry in motor cortex activation reported in brain imaging studies for left-handers relative to right-handers during motor tasks [36,37].

Our results are partially consistent with those reported previously: Rubichi and Nicoletti used a bimanual Simon task with left and right-handed individuals and reported a large Simon effect in the field of the dominant hand [6]. We used a unimanual Simon task and showed a reduced Simon effect in the field of the responding hand. This previous work offered two possibilities for describing the field of operation of the dominant hand. One was based on spatial location where the dominant hand *usually operates*, and the other was based on the location where the dominant hand operates *at a given moment*. To test whether the asymmetrical Simon effect was due to a hand-centered attentional bias, Rubichi and Nicoletti asked participants to perform the task with their hands crossed. Results showed that for right-handed individuals, under a crossed-hands condition (i.e., the right hand presses the left key) the Simon effect was larger in the left visual field. They concluded that the asymmetry in the Simon effect reflects a hand-centered attentional bias that depends on the location where the dominant hand *operates at a given moment*. That is, this hand-centered attentional bias is the behavioral manifestation of the large cortical representation of the dominant hand in both right- and left-handed individuals [38]. According to this possibility, spatial attention processes related to the dominant hand would be more efficient than those related to the nondominant hand. As proposed, shifts of attention are one way to split the visual field between left and right [16]. Therefore, shifts of spatial attention determine the strength of spatial codes and thus the magnitude of the Simon effect. That is, a larger Simon effect should be observed in the field of the dominant hand. Previous results showing similar asymmetries in the Simon effect under bimanual response conditions were interpreted to be a manifestation of hemispheric differences in orienting of attention [7,39]. Taken together, these results are in agreement with ours in that they show that factors associated with the location of the effectors affect spatial coding. However, the possibility that attention is biased according to the location of the dominant hand cannot account for the results reported here for two reasons: First, in our study the Simon effect was smaller in the field of the responding hand; and second, the reduction of the Simon effect occurred for the dominant and nondominant hand (at least for the right-handed participants).

### The grouping model and the Simon effect

Our results can be interpreted in line with the grouping model developed to account for response-cueing effects [20,21]. According to this model, stimuli and response units use multiple and hierarchical coding schemes. As mentioned in the Introduction, this model accounts for the *same hand* advantage in terms of a preattentive grouping process of response units. That is, visual and response elements are grouped according to low-level factors. In the context of our task, left and right responses are given with left and right fingers of the same hand, and therefore, they are part of the same response unit. Following the grouping model, the presentation of the target in the right visual field automatically activates both response units (left and right fingers of the right hand) because they belong to the same response group. Therefore, the mismatch between stimulus location and response location that characterizes the incompatible Simon condition is substantially reduced.

The general architecture of the grouping model can also account for the results of the left-handers when coupled with the observation that left-handed individuals present a smaller degree of cortical asymmetry in motor tasks [36,37]. That is, for left-handed individuals, grouping of responses based on the location of the responding hand might not be so powerful in reducing the Simon effect considering that left-handers are more symmetrical in motor cortex activation. Thus, it is possible that symmetry in motor cortex activation could maintain “left” and “right” response codes to be equally salient for this group. With similar levels of activation, both left and right response codes are subject to interference from the irrelevant spatial location. Consistent with this possibility is the observation that an imbalance in motor activation can be manifested when left-handers respond with the nondominant (right) hand, which in fact happens. Though speculative, the possibility outlined here based on behavioral observation might guide future brain imaging studies. The present results may be the behavioral manifestation of the interaction between motor and attention networks that might differ in right- and left-handed individuals.

### Methodological implications for the effect of responding hand

The results reported here have important implications for the use of stimulus-response interference tasks to address the neural basis of perception-action coding in neurological patients. Mostly bimanual versions of the Simon task have been used to study cognitive control functions in patients with Huntington disease, with both behavioral [40] and fMRI protocols [41]. However, in the last several years, a number of studies have used unimanual versions of stimulus-response inference tasks in patients with cortical and subcortical lesions [42–44]. These studies used a unilateral version of the flanker task, which shares features with the Simon task and, therefore, is subject to the same methodological considerations. Interestingly, differences between left and right visual fields have been reported in a unilateral version of the flanker task following lesions to cortical and subcortical areas [43]. Given the results reported here, it is possible that differences between left and right visual fields might be contaminated by response-related factors. Therefore, our results highlight the need to take into consideration, for both patients and control groups, the analysis of handedness and responding-hand location as a function of visual field. In addition, it is important that controls and patients are matched regarding the responding hand set-up. Note that in a number of these studies, control data on neurologically healthy individuals is not provided; therefore, the impact of task-related factors cannot be estimated.

## Conclusions

Our results show the impact of handedness and responding hand on the Simon effect. At the theoretical level, these findings are in general agreement concerning the importance of attention mechanisms in stimulus-response coding. In addition, they reflect that stimulus-response interference is influenced by multiple and hierarchical coding principles. Finally, our results may foster interesting lines of research when integrated with recent findings showing that the Simon effect is modulated by the presence of task-irrelevant salient events: Dolk and colleagues used a go/nogo Simon task requiring participants to respond to a lateralized auditory stimulus by pressing a single key using the right hand [45]. Across different experiments, an object (e.g., a Japanese waving cat, a clock) was positioned on the participant’s left side. Results showed that even though the participant’s action reference was right (hand position), the presence of a competing event on the participant’s left side produced a leftward representation, resulting in a Simon effect. That is, including additional events to the experimental setting influenced the relevance of spatial locations. Future research is needed to address how the presence of an irrelevant event interacts with specific stimulus-response mappings in the Simon task.

These findings highlight a number of methodological aspects (e.g., handedness, responding hand, and visual field) that need to be taken into consideration when using different response set-ups for examining spatial coding and cognitive control functions in neurological populations.
